# Management of Menstrual Disorder in Adolescent Girls with Intellectual Disabilities: A Blessing or a Curse?

**DOI:** 10.1155/2018/9795681

**Published:** 2018-07-11

**Authors:** Abu Ishak Nurkhairulnisa, Kah Teik Chew, Ani Amelia Zainudin, Pei Shan Lim, Mohamad Nasir Shafiee, Nirmala Kampan, Wan Salwina Wan Ismail, Sonia Grover, Abdul Ghani Nur Azurah

**Affiliations:** ^1^Department of Obstetrics & Gynaecology, Universiti Kebangsaan Malaysia Medical Centre, Kuala Lumpur, Malaysia; ^2^Department of Psychiatry, Universiti Kebangsaan Malaysia Medical Centre, Kuala Lumpur, Malaysia; ^3^Department of Gynaecology, Royal Children's Hospital, Melbourne, VIC, Australia

## Abstract

**Objective:**

This study aims to describe the menstrual pattern and menstrual care of girls with intellectual disabilities and to evaluate the impact of menstruation and awareness of parents/guardians on girls with intellectual disabilities.

**Methodology:**

Parents/guardians of girls aged 9–17 years with known intellectual disabilities who attended a scheduled public forum and Paediatrics and Adolescent Gynaecology Clinic (PAC) were recruited in a questionnaire-based study.

**Results:**

A total of 123 parents/guardians with a mean age of 41.83 ± 5.45 years completed the questionnaire. The mean age of girls with intellectual disabilities was 12.28 ± 2.78 years, and the mean menarcheal age was 11.12 ± 1.76 years. Only 53 (43.1%) parents/guardians were aware of availability of menstrual suppression. Parents/guardians with lower family income (OR = 0.00; 95% CI = 0.00–0.20), unable to manage menses (OR = 0.03; 95% CI = 0.00–0.61), and moderate severity of menses (OR = 0.01; 95% CI = 0.00–1.21), were associated with seeking medical help on menstrual suppression. The factors associated with parents/guardians requesting for sterilization were lower family income (OR = 0.02; 95% CI = 0.00–0.36) and concern about sexual abuse (OR = 0.25; 95% CI = 0.06–0.39).

**Conclusion:**

Menstrual pattern in girls with intellectual disabilities is similar to those without disabilities. Parents/guardians' knowledge and awareness on menstrual suppression were still lacking.

## 1. Introduction

Most girls with intellectual disability attain menarche at the usual age and go through regular menses as their nondisabled peers. A few studies have reported similar age in menarche in comparison to the general population [1–3]. Despite the similarity, anxiety among parents/guardians is high in regard to the impact of menses on the wellbeing of their incapable children [4]. They are particularly concerned about their daughters' capability to handle with menstruation and the increasing risk of sexual abuse. Although the impact of menstruation on the girls and their caretakers is significant, not many of them come forward to seek medical help regarding menstrual management. Most of them perceived that their daughter's menses problem does not affect their daily activities [5].

Literature elaborating menstruation problems among girls with intellectual disabilities is limited especially in Malaysia. Studies revealed that girls with intellectual disabilities often experienced menstruation as a burden to themselves and also to their caretakers [6–8]. Issues that concerned them were pain during menses, embarrassment, lack of privacy, and dealing with the menstrual flow [7]. Anxiety, diffident, and confusion resulting from the insufficient information among them were also been reported [9].

The main purpose of the present study was to examine the menstrual pattern of girls with intellectual disabilities and also to evaluate the impact of menses on these girls and their parents/guardians. It is also aimed at exploring the awareness of menstrual suppression among parents/guardians of these girls and the associated factors that influenced their decision of menstrual suppression methods.

## 2. Materials and Methods

A total of 123 parents/guardians whose daughters were aged between 9 and 17 years with intellectual disabilities were approached and completed the questionnaire. Of these, 72 parents/guardians were from a public forum titled “Menstrual Management in Adolescents with Intellectual Disability” held in May 2012, and 51 parents/guardians were from adolescent gynaecology or paediatric psychiatry clinic at Universiti Kebangsaan Malaysia Medical Centre. All parents/guardians were interviewed by the researchers face to face with a set of validated questionnaire. The permission to use the questionnaire has been granted by the respective author [4].

## 3. Results

### 3.1. Demographic Characteristic

The majority of the respondents were female (91.1%) with a mean age of 41.83 ± 5.45 years. The majority were Malays (58.7%), and 40.7% of the respondents had education level up to secondary school. Approximately one-third of them (33.3%) were from the professional group, and 32.5% were housewife. The majority (52%) have moderate monthly income (RM3001–5000).

The age of the girls ranged from 9 to 17 years with a mean age of 12.28 ± 2.78 years. The type of intellectual disabilities includes Down syndrome (*n*=33, 26.8%), autism (*n*=24, 19.5%), cerebral palsy (*n*=15, 12.2%), and others (*n*=51, 41.5%). Girls in the other groups were mostly slow learners. More than half (64.2%) of the girls attended formal education with only three (2.4%) without education. The degree of disability was assessed by their need for assistance, and half of them (52%) had mild disability, 25.2% moderate disability, and 22.8% severe disability.

At the time of the survey, 12 girls (9.8%) were premenarcheal and therefore could not complete specific questions relating to their menses. A total of 111 girls (90.2%) had attained menarche, and the mean age of menarche was 11.12 ± 1.76 years. The majority (54.1%) had regular menses, and most of them (66.7%) had a menses cycle of 21 to 34 days. Hundred of the girls (90.9%) had menstrual flow lasting for 3 to 7 days. On every menstrual cycle, 91% (101) of them had 1–3 days of heavy menses flow and only two (1.8%) had 4–6 days of heavy menses flow. More than half of the girls (50.5%) experienced mild dysmenorrhea, 24.3% experienced moderate dysmenorrhea, and 25.2% had no dysmenorrhea.

### 3.2. Menstrual Care of Girls with Intellectual Disabilities

From 111 girls who attained menarche, 47 (42.3%) of them were able to manage themselves without help during menses, 46 (41.4%) required parents/guardians help, and 18 (16.2%) were unable to manage themselves at all (Table 1). The parents/guardians were the ones who assisted the girls during their menses (91.9%). Only 24 (21.6%) of the parents/guardians sought medical help to control the girls' menses after they attained menarche. The mean age of the girls during medical advice was 10.57 ± 2.33 years. Factors such as parents/guardians with lower family income (OR = 0.00; 95% CI = 0.00–0.20), unable to manage menses (OR = 0.03; 95% CI = 0.00–0.61), and moderate severity of the girls' menses (OR = 0.01; 95% CI = 0.00–1.21) were statistically significantly associated with parents/guardians to seek medical help on menstrual suppression.

Most of the parents/guardians learned about options available to control menses from friends (36.3%) and school (36.3%). Most of them preferred intramuscular Depo-Provera (58.3%) with only one (4.2%) on Mirena. Majority of parents/guardians (91.9%) were concerned that an irresponsible person might take advantage on their daughter. Only eight (7.2%) parents/guardians had requested permanent sterilization as one of the method of menses suppression for their daughter. Logistic regression analysis revealed that parents/guardians with lower family income (OR = 0.02; 95% CI = 0.00–0.36) and concern about sexual abuse (OR = 0.25; 95% CI = 0.06–0.39) were statistically significantly associated with request for sterilization.

### 3.3. Impact of Menstruation on Social Function

A total of 53 girls (47.7%) had discomfort, three of them (2.7%) felt frightened, and three (2.7%) were depressed during menses. Nevertheless, majority of the parents/guardians (75.7%) felt that the menses did not disturb their daughter's daily activities. Only 17 (15.3%) girls were unable to go to school during menses, with median days of absent from school in a month being 3 days (range 1–20 days). The impact on the social function of parents/guardians during their daughter's menstruation was minimal. For majority of them (82%), their daily schedule was not disturbed, 68.5% of them did not require to take leave from work, and 71.2% did not feel depressed when their daughters had menses. Only 9.9% (11) felt that their daughter's menses were troublesome, and 6.3% (7) felt that the menses interfere with the family's usual outing.

### 3.4. Parents/Guardians Awareness on Menstrual Management in Girls with Intellectual Disabilities

Only 53 (43.1%) of the parents/guardians were aware of the availability of menstrual suppression. Almost half of them (45.5%) did not agree with menstrual suppression using medical intervention. Only 26.8% of parents/guardians agreed with menstrual suppression, and the majority (66.9%) of them were not sure which intervention is the most suitable. Only two (1.7%) of them thought that hysterectomy was the best option (Table 1). Twenty-five (20.7%) of them knew that the progesterone intrauterine system was suitable medical intervention. Most of the parents/guardians (96.7%) were concerned about their daughter's menstruation, and 59.3% (*n*=73) were aware that they could get help on menstrual care. Unfortunately, only 22.8% (*n*=28) had received consultation on menstrual care, and the remaining 77.2% (*n*=95) did not come forward. Most of the parents or guardians received consultation from doctors (71.4%), and another 24.2% received consultation from friends.

The logistic regression test revealed that factors such as parents/guardians' educational level, family income, and severity of menses were statistically significant and associated with awareness of parents/guardians on menstrual suppression in girls with intellectual disability (Table 2). Interestingly, parents/guardians with low educational level (OR = 0.07; 95% CI = 0.01–0.39) and those who had low family income (OR = 0.04; 95% CI = 0.01–0.27) were more inclined to be aware regarding menstrual suppression.

## 4. Discussion

Menstrual distress is frequently reported in girls with intellectual disability and often has a negative impact on the parents/guardians as well as young women themselves. In this study, we discovered that girls with intellectual disability attained menarche at a mean age of 11.12 ± 1.76 years. This finding correlates with the results in the earlier studies [1, 2, 10, 11].

In this study, we found that about 47.7% of the respondents claimed that their daughters experienced pain or discomfort before menses with 50.5% having mild dysmenorrhea. This was consistent with the result from a study conducted among Caucasian women with intellectual disability. The researchers found that abdominal cramps/discomfort was the most severe and prominent symptom during the menses [12]. However, we could not conclude that other symptoms such as nausea and vomiting, diarrhea, headache, and backache were uncommon. This could be due to limitation in conveying the message by the girls which results in misinterpretation by their parents/guardians [13].

Degree of functional status such as physical incapability which requires assistance in activities of daily living such as feeding, dressing, toileting, and menstrual care has been proven to be strongly correlated with parents/guardians wiliness to seek advice regarding menstrual management [4, 5]. We found that the impact of menstruation on parents/guardians and on the girls themselves was not that alarming among the respondents. Even though they had dysmenorrhea, the girls could still go to school. Almost half of the girls were able to manage their menses without help which was similar to a studies conducted in India [14, 15]. This might be because most of the girls had moderate severity of menses which was manageable, and this was the reason why not many parents/guardians stepped forward for advice to control their daughter's menses.

Interestingly, we found that parents/guardians with lower family income were significantly more aware of getting help regarding menstrual suppression compared to the higher family income group. This was a totally opposite result compared to a previous study [5]. This may be related to the availability of information on menstrual care in the Internet that the parents/guardians with higher income did not seek help. Parents/guardians with girls who had moderate severity and who were unable to manage her menses were also keen to seek medical help on menstrual suppression. One-fifth (21.6%) of the parents or guardians had sought medical help, of them half (58.3%) of the parents or guardians had used Depot medroxyprogesterone acetate (DMPA) and one-third (37.5%) of the parents or guardians had used nonsteroidal anti-inflammatory drugs (NSAIDS). The clinical management options for girls with intellectual disabilities include advice, reassurance, medication (oral contraceptive pill, NSAIDS, and DMPA), and surgical options [16].

Parents/guardians requested for sterilization for their daughter were from lower family income group who concerned about sexual abuse. This might be due to higher expenses in buying sanitary pads and pampers if the girl's menstrual problem were remarkable. Usually, this group resides in congested homes which might increase the possibility of sexual abuse. We found that the misunderstanding of hysterectomy as the best option for menstrual suppression was not as alarming as previous study [17]. Hysterectomy involved a lots of potential medicolitigation and ethical issues, which need to be discussed in details with parents/guardians beforehand [18]. The easily available medical counselling for parents/guardians regarding menstrual suppression for girls with learning disability could be the main factor for the finding.

Most of the parents/guardians were concerned about an irresponsible person taking advantage on their daughters. The majority of them were not been given adequate information regarding the menstrual care of girls with intellectual disability. This results in shock, distress, and inappropriate behaviours among girls with intellectual disability when they attain menarche [8, 9]. Hence, it is important for parents to provide such information to their daughters before the beginning of their menses. The most effective way is for the mothers to share their experiences in handling menses with their growing girl, particularly those with intellectual disability [19]. We found that more than half of the parents (78.4%) had not sought medical help on menstrual management for their daughters. They preferred to keep quiet and presumed that their daughter could manage the menses very well. Only 21.6% of them shared their concerns and worries with their friends, teachers, relatives, and doctors.

Our findings suggested that the parents/guardians were lacking information on menstruation in girls with intellectual disability. Very often, most of the parents have the perception that it is too early for them to worry about the menses of their intellectual disability daughter. Hence, they feel that there is no urgency to ask for advices regarding menses management for their daughter. Furthermore, poor skills and knowledge among healthcare providers in handling with girls with learning disability may discourage parents to seek advices and information [20]. Social taboo and cultural and religional belief may be the factors that prevent parents to ask for help from doctors.

There were few limitations in this study. The sample size in this study was small as it was conducted in a tertiary centre, so the findings should not represent the whole community of Malaysia. Besides that, only the parents/guardians' opinion was taken into account in this study. Nevertheless, the results had provided us a better understanding on menstrual care among girls with intellectual disability in Klang Valley. This research suggested that menstruation was not as problematic as we thought for girls with intellectual disabilities.

## 5. Conclusion

The finding from this study suggests that parents/guardians' knowledge and awareness on menstrual management or suppression are still lacking. Providing disabled girls, their family's members, and their caretakers with adequate information enables the least restrictive and least invasive approach to health matters. Understanding the impact of menstruation on young women with intellectual disabilities and also their caretakers may assist clinicians in caring for these young women. Healthcare providers need to be educated on these issues and encouraged to make appropriate referral to ensure better care is provided to these young women. Further studies are needed to look into the matter especially in those who do not have access to health services.

## Figures and Tables

**Table 1:** Menstrual care for girls with intellectual disabilities.

	*n*	%
Ability to manage menses		
Yes, without help	47	42.3
Yes, with help	46	41.4
Not at all	18	16.2

Person responsible in assisting the girl during menses		
Yourself	90	81.1
Your partner	12	10.8
Relatives	6	5.4
Others	3	2.7

Ever sought medical help		
Yes	24	21.6
No	87	78.4

Attained menarche at the time of seeking medical help		
Yes	24	100.0
No	0	0

Source of information		
School	8	36.3
Doctors	1	4.5
Relatives	5	22.7
Friends	8	36.3
Books	0	0
Internet	0	0

Type of medical treatment used		
Pain killer	9	37.5
Depo	14	58.3
Progesterone intrauterine system	1	4.2
COCP	0	0
Hysterectomy	0	0

Concerns about sexual abuse		
Yes	113	91.9
No	10	8.1

Ever requested for sterilization		
Yes	8	7.2
No	103	92.8

**Table 2:** Factors associated with awareness of parents/guardians on menstrual suppression in girls with intellectual disabilities.

	*B*	SE	Significance	OR	95% CI		*R* ^2^
					Lower	Upper	
Parents/guardians educational level	−2.60	0.85	0.00	0.07	0.01	0.39	0.64
Family income	−3.12	0.93	0.00	0.04	0.01	0.27	
Severity of menses	1.42	0.66	0.03	4.12	1.12	15.16	
Functional status	0.74	0.84	0.38	2.09	0.40	10.81	
Ability to manage menses	0.30	1.06	0.78	1.35	0.17	10.74	
Concerns on sexual abuse	−1.51	1.39	0.28	0.22	0.01	3.38	
School abstinence	−1.19	1.03	0.25	0.30	0.04	2.29	
Constant	−21.57	9075.93	1.00	0.00	—	—	

## Data Availability

All data in this research belong to the copyright of Universiti Kebangsaan Malaysia Medical Centre.
